# A comparison of two different software packages for analysis of body composition using computed tomography images

**DOI:** 10.1016/j.nut.2018.06.003

**Published:** 2019-01

**Authors:** Katie E Rollins, Amir Awwad, Ian A. Macdonald, Dileep N. Lobo

**Affiliations:** a Gastrointestinal Surgery, Nottingham Digestive Diseases Centre, National Institute for Health (NIHR) Research Nottingham Biomedical Research Centre, Nottingham University Hospitals and University of Nottingham, Queen's Medical Centre, Nottingham United Kingdom; b Sir Peter Mansfield Imaging Centre (SPMIC), University of Nottingham, University Park, Nottingham, United Kingdom; c School of Life Sciences, University of Nottingham, Queen's Medical Centre, Nottingham, United Kingdom; d MRC/ARUK Centre for Musculoskeletal Ageing Research, School of Life Sciences, University of Nottingham, Queen's Medical Centre, Nottingham United Kingdom

**Keywords:** Computed tomography, Body composition, Sarcopenia, Myosteatosis, OsiriX, SliceOMatic

## Abstract

**Objectives:**

The analysis of body composition from computed tomography (CT) imaging has become widespread. However, the methodology used is far from established. Two main software packages are commonly used for body composition analysis, with results used interchangeably. However, the equivalence of these has not been well established. The aim of this study was to compare the results of body composition analysis performed using the two software packages to assess their equivalence.

**Methods:**

Triphasic abdominal CT scans from 50 patients were analyzed for a range of body composition measures at the third lumbar vertebral level using OsiriX (v7.5.1, Pixmeo, Switzerland) and SliceOmatic (v5.0, TomoVision, Montreal, Canada) software packages. Measures analyzed were skeletal muscle index (SMI), fat mass (FM), fat-free mass (FFM), and mean skeletal muscle Hounsfield Units (SMHU).

**Results:**

The overall mean SMI calculated using the two software packages was significantly different (SliceOmatic 51.33 versus OsiriX 53.77, *P* < 0.0001), and this difference remained significant for non-contrast and arterial scans. When FM and FFM were considered, again the results were significantly different (SliceOmatic 33.7 versus OsiriX 33.1 kg, *P* < 0.0001; SliceOmatic 52.1 versus OsiriX 54.2 kg, *P* < 0.0001, respectively), and this difference remained for all phases of CT. Finally, when analyzed, mean SMHU was also significantly different (SliceOmatic 32.7 versus OsiriX 33.1 HU, *P* = 0.046).

**Conclusions:**

All four body composition measures were statistically significantly different by the software package used for analysis; however, the clinical significance of these differences is doubtful. Nevertheless, the same software package should be used if serial measurements are being performed.

## Highlights

• We clarify the equivalence of body composition analysis from computed tomography images using two different software packages.
• Analysis was performed using SliceOmatic and OsiriX packages on 50 patients who had undergone triphasic scans.
• Body composition measures were significantly different between the two software packages, but the clinical significance of these is doubtful.
• We recommend that for serial body composition analysis and for comparative purposes, the software package employed should be consistent.

## Introduction

Computed tomography (CT) analysis of body composition to measure fat mass (FM) and fat-free mass (FFM), to calculate skeletal muscle index (SMI), and to diagnose sarcopenia and myosteatosis has become increasingly common, with literature now linking sarcopenia and myosteatosis with reduced overall survival [1], [2], decreased tolerance to chemotherapy [3], [4], and increased complications [5], [6] after surgery in patients presenting with various types of malignancy.

However, the methodology for calculating body composition from CT images is variable between studies, from the nature of the CT scan used including the vertebral level, to the use of contrast medium, to the software used to perform the analysis. The effects of the use of contrast medium in CT scanning in body composition analysis has previously been recognized to have a significant effect on results, especially the diagnosis of myosteatosis [7], [8]. Despite these inconsistencies in analysis, the results of these studies are used interchangeably, with the definition of neither sarcopenia or myosteatosis stipulating any conditions about how these derived values are calculated.

There are currently two software packages used commonly to analyze body composition from CT: SliceOmatic (TomoVision, Montreal, Canada) and OsiriX (Pixmeo, Switzerland), the results of which are also used interchangeably. One study in patients with rectal cancer [9] suggested that SliceOmatic, ImageJ (National Institutes of Health, Bethesda, MD, USA), FatSeg (Biomedical Imaging Group Rotterdam of Erasmus MC, Rotterdam, The Netherlands, using MeVisLab [Mevis Medical Solutions, Bremen, Germany]) and OsiriX analysis provide excellent levels of agreement. However, the study [9] did not consider mean skeletal muscle Hounsfield Unit (SMHU) as a surrogate for myosteatosis. The aim of the present study was to compare the SliceOmatic and OsiriX software packages and determine if there was a difference in calculated measures of body composition, namely SMI, FM, FFM and mean SMHU, using CT images.

## Methods

In a single-center retrospective study, CTs from 50 patients who underwent triple-phase abdominal scans (non-contrast, arterial, and portovenous phases) between April 2014 and September 2015 were analyzed using two different software packages: SliceOmatic v5.0 and OsiriX v7.5.1. The patients were initially identified retrospectively from the Computerised Radiology Information System (CRIS v 2.09, HSS, Healthcare Systems, Mansfield, UK). The underlying pathology necessitating the CT was variable, and included trauma, suspected intraabdominal or gastrointestinal bleeding, pancreatic or hepatic pathology, and renal lesions. Three axial slices were selected from each triphasic abdominal CT (total analyzed slices in the study = 50 × 3 = 150 slices). Each slice was anatomically localized using coronal and sagittal multiplanar reformats (MPRs) to ensure it specifically lies at the third lumbar vertebra (L3). Slices were analyzed as Digital Imaging and Communication in Medicine (DICOM) images obtained from a picture archiving and communication system (PACS). Electronic data were collated for patient demographic characteristics, including height and weight data from within 1 mo of the date of the CT.

### Scan acquisition

During the study period, two CT scanners were in use at Nottingham University Hospitals NHS Trust where the study was conducted. The first was Ingenuity 128 (Phillips Healthcare, Best, The Netherlands) and the second was Optima CT660 (GE Healthcare, Milwaukee, WI, USA). These were calibrated weekly to ensure that quality assurance testing was met for the Hounsfield Unit (HU) density of air (HU = –1000) and water (HU = 0). Arterial and portovenous phase scans were obtained using intravenous (IV) administration of contrast medium (100 mL fixed dose of Iopamidol, Niopam 300, Bracco, Buckinghamshire, UK). The timings of different phase scans were standardized, first with an unenhanced scan, then the arterial phase performed at 10 to 20s, and finally the portovenous scan at 65s.

### Body composition analysis

The three phases of CT slice on each individual patient were analyzed by a single observer, our group having previously established high rates of interobserver reliability (SMI *r*^2^ = 0.975, *P* < 0.0001; mean SMHU *r*^2^ = 0.965, *P* < 0.0001) in the analysis of body composition variables using the techniques adopted in this study [7]. The software packages, SliceOmatic and OsiriX, were each used to calculate the cross-sectional area of skeletal muscle, visceral and subcutaneous/intramuscular adipose tissue. The different tissue types were identified by their differing radiodensities; skeletal muscle of –29 to +150 HU, visceral adipose of –150 to –50 HU and subcutaneous/intramuscular adipose of –190 to –30 HU. The mean SMHU density was also recorded for all scans analyzed.

Previously described regression equations for the calculation of whole-body FM and FFM from a single cross-sectional CT slice were used [10]:

$$\text{Total}\;\text{body}\;\text{FM}\;\left(\text{kg}\right)=0.042\times [\text{total}\;\text{adipose}\;\text{tissue}\;\text{area}\;\text{at}\;L3\left(cm^{2}\right)]+11.2$$

$$\text{Total}\;\text{body}\;\text{FFM}\;\left(\text{kg}\right)=0.3\times [\text{total}\;\text{skeletal}\;\text{muscle}\;\text{area}\;\text{at}\;L3\;\left(cm^{2}\right)]+6.06$$

The cross-sectional area of skeletal muscle was also transformed into the SMI by modifying it by patient height.

### Statistical analysis

Statistical analysis was performed using SPSS v 22 (IBM, SPSS Statistics, Armonk, NY, USA) and GraphPad Prism v 6.0 (GraphPad, La Jolla, CA, USA). FM, FFM, SMI, and mean SMHU density values, with data checked for normality using the D'Agostino–Pearson normality test. Data were compared between different software packages using the Student's paired *t* test when normality was confirmed, and the Wilcoxon matched-pairs signed rank test when the data were not distributed normally. Pearson's coefficient of correlation was used to compare the body composition values calculated from the two different software packages and Bland-Altman plots used to reveal any systematic error between the analyses. All analyses were performed using two-tailed testing with a significance level set at *P* < 0.05.

## Results

Of the 50 patients included during the study period from April 2014 to September 2015, there were 33 men and 17 women, with a mean body mass index (BMI) of 30.4 kg/m^2^ (SD 4).

### Skeletal muscle index

Analysis of body composition by OsiriX gave a significantly greater value for SMI than scans analyzed using SliceOmatic (53.8 versus 51.3 cm^2^/m^2^, *P* < 0.0001) on Wilcoxon matched-pairs signed rank test, performed according to the D'Agostino–Pearson test, demonstrating a lack of normality in the data from OsiriX analysis (*K*^2^ = 7.831, *P* = 0.012). This difference remained between scans analyzed in non-contrast and arterial phases; however, there was no difference in scans analyzed in the portovenous phase (Table 1).

**Table 1:** Comparison of body composition measures calculated by OsiriX vs SliceOmatic software packages in non-contrast, arterial, and portovenous phase scans

	Non-contrast phase scan	Arterial phase scan	Portovenous phase scan
Skeletal muscle index (cm^2^/m^2^) ± SD			
SliceOmatic	51 ± 10.1	51.4 ± 10.1	51.6 ± 9.9
OsiriX	53.3 ± 10.4	53.6 ± 11.1	54.4 ± 10.7
Mean difference between modalities	–2.3 ± 2.2	–2.2 ± 3.3	–2.7 ± 3
*P*-value	<0.0001	<0.0001	0.189
Fat mass (kg)			
SliceOmatic	34.1 ± 9.1	33.7 ± 8.9	33.5 ± 9
OsiriX	33.4 ± 9	33 ± 8.7	32.8 ± 9
Mean difference between modalities	0.7 ± 0.6	0.7 ± 0.8	0.7 ± 0.5
*P*-value	<0.0001	<0.0001	<0.0001
Fat-free mass (kg)			
SliceOmatic	51.8 ± 11.3	52.1 ± 11.3	52.3 ± 11.3
OsiriX	53.9 ± 11.7	54.1 ± 12.1	54.8 ± 11.9
Mean difference between modalities	–2.1 ± 2	–2 ± 2.9	–2.4 ± 2.7
*P*-value	<0.0001	<0.0001	<0.0001
Mean Skeletal Muscle Hounsfield Units			
SliceOmatic	30.1 ± 9.3	33 ± 9.9	35.4 ± 10.2
OsiriX	30.6 ± 8.6	32.7 ± 9.4	35.7 ± 10
Mean difference between modalities	–0.5 ± 2.2	0.3 ± 2.1	–0.2 ± 2.4
*P*-value	0.120	0.213	0.450

**Fig. 1:**
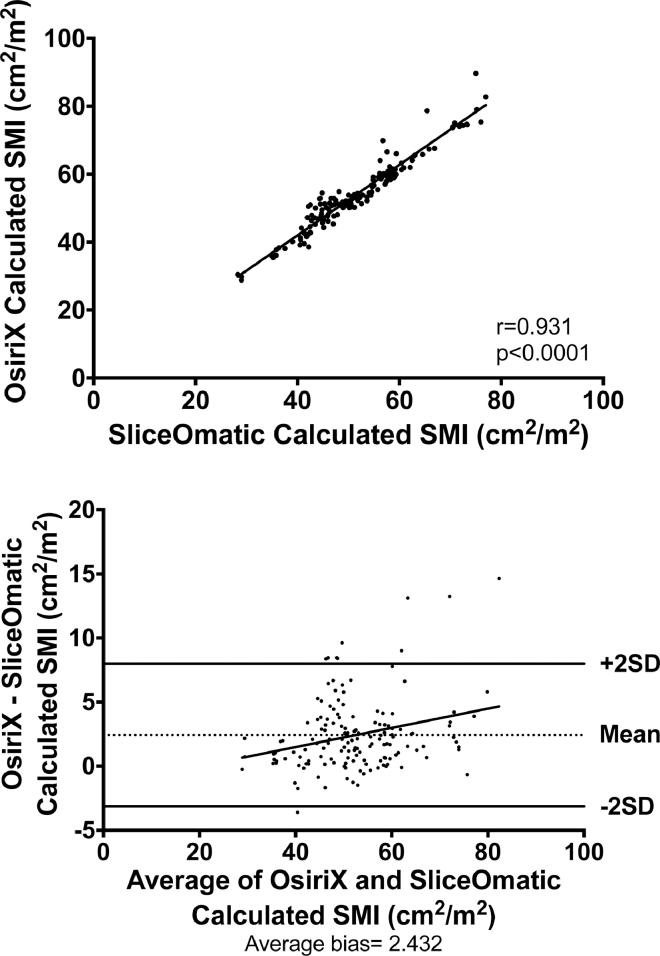
Correlation between mean skeletal muscle index (SMI) calculated using OsiriX and SliceOmatic software packages and Bland-Altman plots to assess for systematic bias.

There was a significant positive correlation in SMI between analysis conducted using OsiriX and SliceOmatic software (*r* = 0.965, *P* < 0.0001) and evidence of a positive systematic bias on Bland -Altman testing (average bias = 2.432; Fig. 1).

**Fig. 2:**
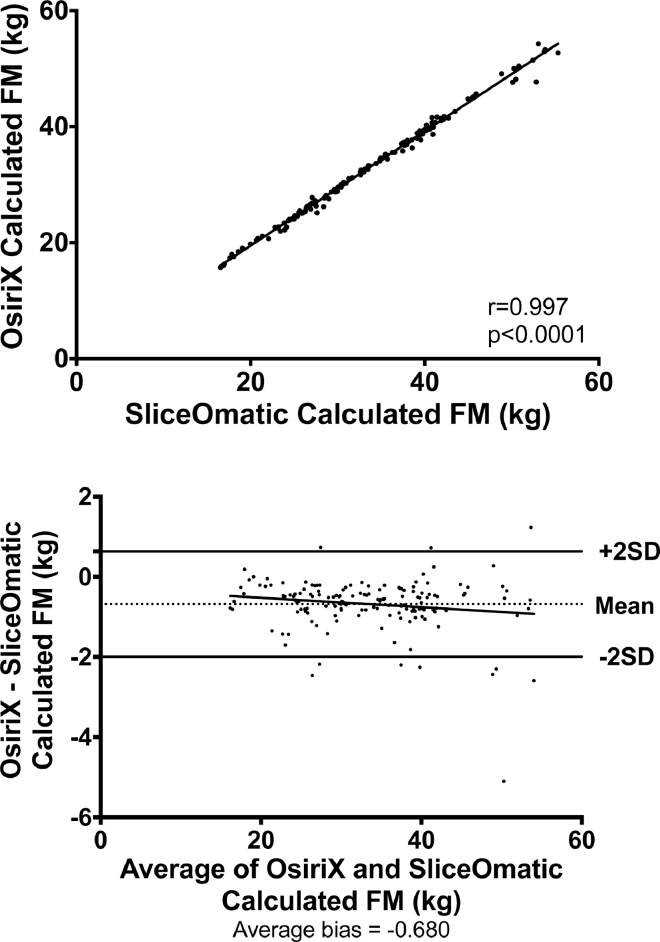
Correlation between fat mass (FM) calculated using OsiriX and SliceOmatic software packages and Bland-Altman plots to assess for systematic bias.

**Fig. 3:**
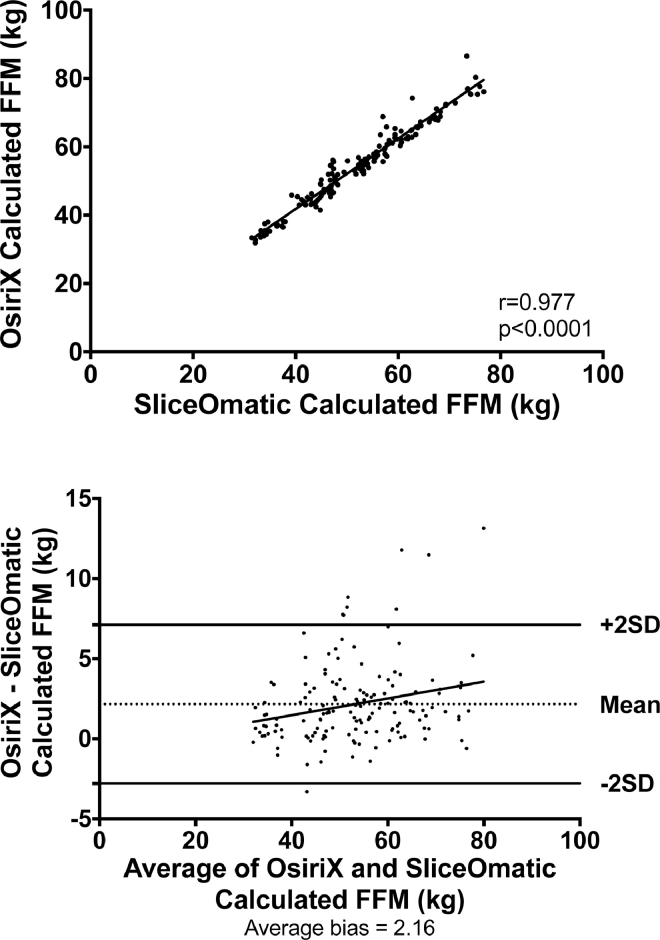
Correlation between fat-free mass (FFM) calculated using OsiriX and SliceOmatic software packages and Bland-Altman plots to assess for systematic bias.

**Fig. 4:**
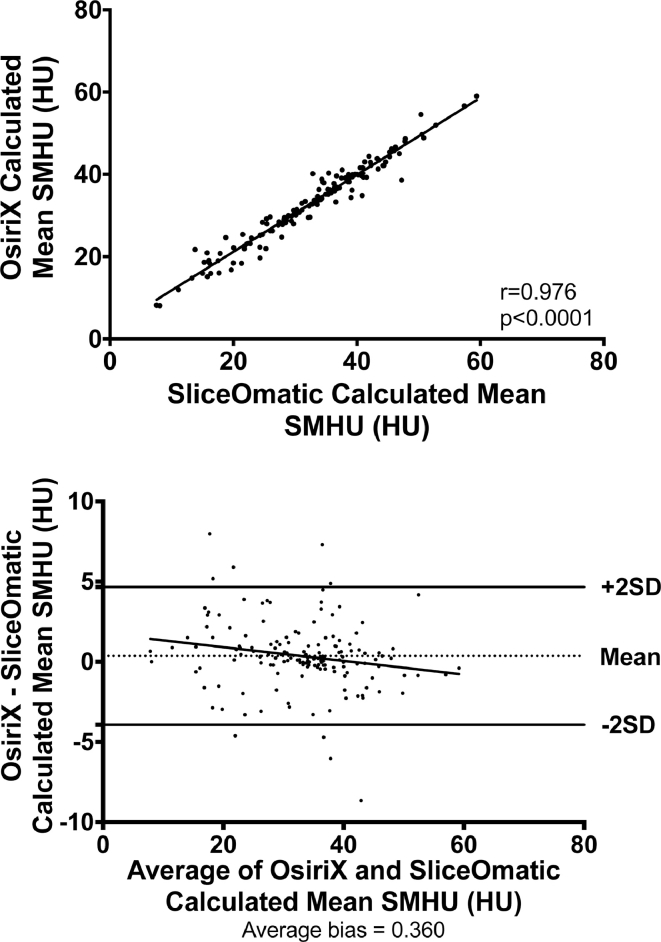
Correlation between mean skeletal muscle Hounsfield Units (SMHU) calculated using OsiriX and SliceOmatic software packages and Bland-Altman plots to assess for systematic bias.

### Fat mass

FM calculated by OsiriX was significantly lower than that calculated by SliceOmatic (33.1 versus 33.7 kg, *P* < 0.0001) as calculated by the Student's paired *t* test, as the data were demonstrated to be normally distributed, and this difference was seen when all individual phase data were analyzed (Table 1).

The correlation between FM analysis using OsiriX and SliceOmatic was significant (*r* = 0.997, *P* < 0.0001) and Bland-Altman testing revealed no evidence of a systematic bias (average bias = –0.680; Fig. 2).

### Fat-free mass

Analysis of FFM using the two software packages demonstrated significantly greater values with OsiriX analysis than with SliceOmatic (54.2 versus 52.1 kg, *P* < 0.0001) as calculated by the Student's paired *t* test as the data were demonstrated to be normally distributed. This finding remained consistent in slices analyzed in non-contrast, arterial, and portovenous phases (Table 1).

There was a significant positive correlation between analysis of FFM performed using OsiriX and SliceOmatic software packages (*r* = 0.977, *P* < 0.0001) and there was evidence of a systematic bias on Bland-Altman testing (average bias = 2.16; Fig. 3).

### Mean skeletal muscle Hounsfield units

The mean SMHU density was overall significantly higher when analyzed using OsiriX rather than SliceOmatic software (33.1 versus 32.7 HU, *P* = 0.046) as calculated by the Student's paired *t* test as the data were demonstrated to be normally distributed. However, when the individual phases of CT were compared, there were no significant differences between OsiriX and SliceOmatic (Table 1).

There was a significant positive correlation in the mean SMHU between the two software packages (*r* = 0.976, *P* < 0.0001) and no evidence of any systematic bias (average bias = 0.360; Fig. 4).

## Discussion

This study provides evidence of the relative clinical equivalence of analysis of body composition measures analysed by two different software packages, namely OsiriX and SliceOmatic. However, statistically significantly greater SMI, FFM, and mean SMHU values and significantly lower FFM were demonstrated when the analyses were performed with OsiriX compared with SliceOmatic. There was significant positive correlation for all measures when the two software packages were compared, although Bland-Altman testing revealed evidence of a significant systematic bias when analyzing SMI and FFM. The results of the present study are similar to those of the previously published comparison of OsiriX, SliceOmatic, ImageJ, and FatSeg [9], which found that body composition in terms of cross-sectional muscle area, visceral adipose tissue area, and subcutaneous adipose tissue area had excellent levels of agreement, suggesting that the results of analysis with the different software packages could be used interchangeably. However, this study suggested evidence of a systematic bias in the analysis of SMI and FFM, which should be considered when comparing results of body composition analysis performed using different software packages. The previous study [9], however, did not include myosteatosis, as calculated by the mean SMHU value, which is being increasingly used in body composition analysis. Additionally, the present study considered the different phases of abdominal CT (non-contrast, arterial, and portovenous), which were not considered by the previous literature; indeed no statement is made regarding the phase of CT scan considered by the previous study [9].

Although the results of the present study demonstrate statistically significant differences in body composition variables by software package used for analysis, the clinical significance of several of these outcomes is doubtful. The mean SMHU was different by just 0.4 HU, much less than the difference in SMHU between different phases of CT scan (in OsiriX analysis a difference of 5.1 HU was seen between non-contrast and portovenous scans and 5.3 HU in SliceOmatic analysis). This discrepancy in radiodensity of skeletal muscle has been documented previously [7] and its clinical relevance questioned. Therefore, with such a small difference, this is very unlikely to have a significant effect on the diagnosis of myosteatosis. Similarly, the difference between software packages was minimal in FM analysis, with an overall difference of 0.7 kg, which represents just 1.8% of the overall mass from OsiriX analysis. The difference was more pronounced in SMI and FFM analysis, with a difference of 2.5 cm^2^/m^2^ (4.6%) and 2.1 kg (3.9%) respectively, which are more likely to represent a clinically relevant difference. This difference in body composition variables has not been demonstrated previously, and the results of body composition analysis using OsiriX and SliceOmatic software packages are used interchangeably within the literature.

This study was conducted retrospectively. However, all scans were performed on individual patients at the same time, so although the hydration status was not known, it would be consistent for all scans and, therefore, would not have an effect on these results. Height and weight data were not always available from the date of the scan, which may render the calculation of body composition measures less accurate.

Further work on body composition analysis is necessary to standardize the methodology used to calculate clinical body composition outcomes including the presence of sarcopenia and myosteatosis. This should include muscle biopsy samples of the rectus abdominis at the L3 vertebral level to correlate radiologic and histologic analysis of skeletal muscle.

To our knowledge, this is the first study to investigate the analysis of body composition variables including myosteatosis by software package of analysis, and has demonstrated statistically significant differences in values in all outcomes. Although some statistically significant differences were demonstrated between the two software packages, these are unlikely to be clinically relevant. However, given the demonstrable differences in body composition measures, it is suggested that the two packages should not be used interchangeably for clinical or research purposes.
